# Magnetic resonance imaging of anal cancer: tumor characteristics and early prediction of treatment outcome

**DOI:** 10.1007/s00066-023-02114-5

**Published:** 2023-07-10

**Authors:** Bettina A. Hanekamp, Ellen Viktil, Kathinka S. Slørdahl, Johann Baptist Dormagen, Nils E. Kløw, Eirik Malinen, Cathrine Brunborg, Marianne G. Guren, Anselm Schulz

**Affiliations:** 1 Department of Radiology, Oslo University Hospital Ullevål, Oslo, Norway; 2 Institute of Clinical Medicine, University of Oslo, Oslo, Norway; 3 Department of Oncology, Oslo University Hospital Ullevål, Oslo, Norway; 4 Department of Physics, University of Oslo, Oslo, Norway; 5 Department of Medical Physics, Oslo University Hospital, Oslo, Norway; 6 Oslo Centre for Biostatistics and Epidemiology, Research Support Services, Oslo University Hospital, Oslo, Norway

**Keywords:** Anal cancer, Diffusion magnetic resonance imaging, Area under the curve, Biomarker, Chemoradiotherapy

## Abstract

**Purpose:**

To analyze tumor characteristics derived from pelvic magnetic resonance imaging (MRI) of patients with squamous cell carcinoma of the anus (SCCA) before and during chemoradiotherapy (CRT), and to compare the changes in these characteristics between scans of responders vs. nonresponders to CRT.

**Methods:**

We included 52 patients with a pelvic 3T MRI scan prior to CRT (baseline scan); 39 of these patients received an additional scan during week 2 of CRT (second scan). Volume, diameter, extramural tumor depth (EMTD), and external anal sphincter infiltration (EASI) of the tumor were assessed. Mean, kurtosis, skewness, standard deviation (SD), and entropy values were extracted from apparent diffusion coefficient (ADC) histograms. The main outcome was locoregional treatment failure. Correlations were evaluated with Wilcoxon’s signed rank-sum test and Pearson’s correlation coefficient, quantile regression, univariate logistic regression, and area under the ROC curve (AUC) analyses.

**Results:**

In isolated analyses of the baseline and second MRI scans, none of the characteristics were associated with outcome. Comparison between the scans showed significant changes in several characteristics: volume, diameter, EMTD, and ADC skewness decreased in the second scan, although the mean ADC increased. Small decreases in volume and diameter were associated with treatment failure, and these variables had the highest AUC values (0.73 and 0.76, respectively) among the analyzed characteristics.

**Conclusion:**

Changes in tumor volume and diameter in an early scan during CRT could represent easily assessable imaging-based biomarkers to eliminate the need for analysis of more complex MRI characteristics.

## Introduction

Squamous cell carcinoma of the anus (SCCA) is a rare cancer; however, the incidence is increasing [1]. The curative treatment is chemoradiotherapy (CRT) with mitomycin (MMC) and 5‑fluorouracil (5-FU) or capecitabine [2–6]. Although overall survival is good, locoregional recurrence is still of significant concern, especially for patients with locally advanced disease [3, 7, 8]. Treatment of locally recurrent disease is curative-intent salvage surgery, usually with extensive pelvic surgery [9]. CRT is associated with considerable late side effects that have an impact on quality of life [10, 11], and there is a delicate balance between tumor dose escalation and toxicity. There is a need to clarify tumor characteristics and identify prognostic biomarkers to promote the future development of personalized CRT for SCCA to improve outcomes [12].

Magnetic resonance imaging (MRI) and positron-emission tomography computed tomography (PET/CT) are used for diagnosis and staging of SCCA and for radiotherapy treatment planning, and have a role in response evaluation after CRT completion [5]. Considering that advanced pelvic MRI is well established and plays an important role in the clinical workflow for SCCA, it is valuable to investigate whether pelvic MRI may provide imaging-based prognostic biomarkers [13]. As part of the MRI examination of the pelvis in SCCA and rectal cancer, high-resolution T2-weighted sequences (T2W) are used to depict the tumor, tumor size, and anatomic relations [5]. In rectal cancer, extramural tumor depth (EMTD) is an important prognostic parameter [14]. Although rectal cancer and SCCA have very different pathological features despite their anatomic neighborhood, we sought to evaluate this metric for SCCA.

Diffusion-weighted imaging (DWI) enables quantification of the motion of water molecules in tissues. In solid cancers, the free movement of water molecules is often restricted due to high tissue density and interstitial fluid pressure, resulting in a low apparent diffusion coefficient (ADC). In oncology, DWI is used to identify malignant tumors, characterize tumor aggressiveness, and evaluate treatment response [15]. Moreover, identification of an imaging-based early biomarker using an additional second MRI scan during CRT might provide information that has the potential to guide treatment modification. Early identification during CRT of the subgroup of patients at risk of treatment failure would allow us to personalize treatment. Although there are no established alternative personalized modified treatment concepts yet, conceivable trial options could be, for instance, dose escalation or intensified treatment with chemotherapy or immunotherapy.

The use of DWI to predict outcome has been investigated in squamous cell carcinomas of other anatomic sites, such as head and neck squamous cell carcinoma (HNSCC) [16–18] and cervical squamous cell carcinoma [19]. Changes in the ADC between a baseline MRI scan prior to CRT and a second scan in the early phase of radiotherapy (1–3 weeks) were consistently correlated with local control. In addition, there is an increasing number of studies indicating the usefulness of DWI to predict the response to CRT in rectal cancer [20, 21]. There are few studies on MRI-based metrics as biomarkers for SCCA [22–25]. Given the rarity of SCCA, it is challenging to obtain a sample size with sufficient statistical power for biomarker development and validation.

This study aimed to assess MRI-based tumor characteristics of SCCA prior to CRT (baseline scan) and in the early phase of CRT in week 2 (second scan), with the secondary aim of identifying a marker that could be used to predict treatment response.

## Materials and methods

### Patient inclusion

The present investigation is part of the “Anal cancer radiotherapy—prospective study of treatment outcome, patient-reported outcomes, utility of imaging and biomarkers, and cancer survivorship (ANCARAD)” study, a prospective multidisciplinary observational trial (NCT01937780). Histologically proven SCCA, planned CRT, and adequate performance status (ECOG 0–2) were the main inclusion criteria. A total of 141 eligible patients referred to Oslo University Hospital (OUS) between October 2013 and September 2017 were included in the study. The study was approved by the Regional Ethical Committee South–East (2012/2274) and the local data protection officer. All patients provided written informed consent. As part of the study protocol, patients underwent pelvic MRI, CT of the thorax/abdomen/pelvis, and, in most cases, PET/CT prior to CRT for staging. Due to logistical reasons, approximately half of the MRI scans at baseline were performed at OUS with the study protocol. The remaining patients were examined at regional hospitals and institutions with varying MRI protocols and were not included in the current study. A prospective cohort was formed from a subset of patients with baseline scans following the 3T MRI study protocol at OUS; these patients were invited to participate in the study and consented to additional imaging during the second week of CRT (second scan) with pelvic MRI.

**Fig. 1:**
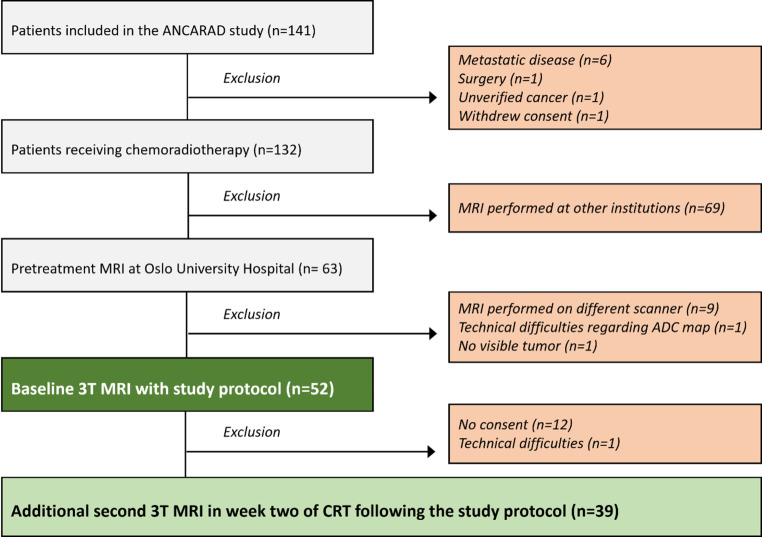
Flowchart showing the inclusion and exclusion of patients in the ANCARAD study and for the present MRI analyses

The total number of patients eligible for a dedicated study 3T MRI scan at OUS was 52 prior to CRT (baseline scan). Of these patients, 39 had an additional second scan during the second week of CRT (Fig. 1).

### Magnetic resonance imaging

All pelvic MRI scans included in this analysis were performed with a 3T Philips Ingenia MRI scanner (Philips Healthcare, Amsterdam, The Netherlands). Patients were scanned in the supine position and a pelvic phased array coil was used. If not contraindicated, all patients received 1 mg glucagon (Glucagon®, Novo Nordisk, Bagsvaerd, Denmark) intramuscularly prior to the examination and 20 mg butylscopolamine (Buscopan®, Opella Healthcare, Gentilly, France) intravenously during the examination to reduce bowel movement artifacts. The MRI study protocol included a combination of T2-weighted imaging (T2WI) and DWI sequences (Table 1). ADC maps were generated using the standard algorithm provided on the console of the scanner using b0 and b1200 values.

**Table 1:** MRI protocol with acquisition parameters for the Philips-Ingenia 3T MR scanner (study protocol)

Parameter	T2WI (oblique axial^a^/axial)	DWI (*b*-values were 0, 50, and 1200 s/mm^2^)
Repetition time (ms)	≥ 3700	2167
Echo time (ms)	80	75
Slice thickness (mm)	3.0/5.0	5.0
Slice gap (mm)	1.0/1.0	1.0
Matrix size	400 × 388/480 × 470	116 × 132
No. averages	1	4
Field of view (mm2)	200 × 200/240 × 240	400 × 279
No. slices	28/35	35

^a^Perpendicular to the long axis of the anal canal and/or tumor

### Chemoradiotherapy

All patients were discussed in a multidisciplinary team (MDT) meeting and treated according to national guidelines. Radiotherapy was delivered using 3D conformal radiotherapy, intensity-modulated radiotherapy (IMRT), or volumetric modulated arc therapy (VMAT). The radiotherapy doses administered to the primary tumor and the metastatic lymph nodes were 54.0 or 58.0 Gy, depending on stage, while the dose to the noninvolved nodal regions was 46.0 Gy. Chemotherapy was delivered with MMC 10 mg/m^2^/day (one patient received cisplatin instead of MMC) on day 1 and 5‑FU 1000 mg/m^2^/day days 1–4, and a new cycle of MMC/5-FU began on day 29 for patients with advanced disease. Further details and the main results of the ANCARAD study have been published previously [26].

### Follow-up

Tumor response was routinely assessed 3 months after CRT by clinical examination, anoscopy/proctoscopy, imaging with pelvic MRI, and either PET/CT or CT thorax/abdomen/pelvis. All patients were followed for at least 5 years or until death or recurrence. Patients who had residual disease or later developed recurrence were considered for salvage surgery. The main outcome in our study was locoregional treatment failure and was defined as failure to demonstrate a complete response (CR) 6 months after CRT or evidence of local or regional disease after CR had been achieved. Patients with locoregional treatment failure were considered for salvage surgery. Using these clinical follow-up data, patients were divided into a (locoregional) failure group or a nonfailure group, depending on the outcome.

### Characteristics, data analysis, and statistics

The MR images were anonymized, and a board-certified radiologist (B.A.H.) with extensive experience in pelvic MRI delineated a region of interest (ROI) encompassing the macroscopic tumor on T2W images together with DW images on a multimodality reading platform (Syngovia VB30®, Siemens Healthineers, Erlangen, Germany). Areas of suspected necrosis were not excluded, and tumor volume was calculated. The greatest dimension (mm) of the tumor, tumor infiltration to other organs and structures, external anal sphincter infiltration (EASI), and extramural tumor depth (EMTD) were assessed on T2W images as part of the MR study protocol and for TNM staging. Similar to measurement in rectal cancer [27], the maximum depth of extramural infiltration was measured from the outer edge of the internal sphincter of the anus/muscularis propria to the outer margin of the tumor (mm). To evaluate ADC values in the tumor, the ROI for each T2W image was propagated to the corresponding ADC map. The mean ADC in the tumor volume was calculated, and skewness, kurtosis, entropy, and standard deviation (SD) were extracted from the ADC histogram analysis. The histogram analysis was executed by LIFEx, a freeware for radiomic feature calculation in multimodality imaging [28].

All statistical analyses were performed using STATA (Statistical Software: Release 16, StataCorp LLC, TX, USA). To assess the differences in MRI characteristics between the two different timepoints (baseline and second scan during CRT), the Wilcoxon signed rank-sum test was used. Pearson’s correlation coefficient between different baseline variables was estimated. The median differences in the relative change of the MR characteristics between the scans were compared between the failure group and the nonfailure group by quantile regression. Univariate logistic regression analysis with estimation of odds ratio (OR) and AUC values from ROC curves was used to assess the association of locoregional failure with the MRI characteristics at baseline scan, at second scan, and the relative change between the scans. Optimal cutoff points from receiver operating characteristic (ROC) curves to predict treatment failure were estimated by the LIU method [29]. *P*-values of  0.05 were considered significant.

## Results

The median age of the 52 patients included in the MRI study was 61 years (range 40–90); 77% were women, 48% had T3–T4 tumors, 58% had N1–N3 disease, and 86% had human papillomavirus (HPV)-positive tumors. Patient characteristics, treatment, and follow-up data corresponded well with data from the main study [26], except for a higher percentage of N1–N3 disease in the MRI study (58% versus 45%). The median follow-up was 60 months (range 5–85).

Treatment failure occurred in 8/52 patients (15%); of these, locoregional failure occurred in 7/52 patients (13%). At baseline MRI, 36/52 patients (69%) had extramural tumor infiltration with a median EMTD of 5 mm, and 29/52 (56%) had external sphincter infiltration. Baseline tumor size characteristics were significantly correlated with each other: the median baseline diameter was 40 mm (range 14–140), and the median volume was 14.5 cm^3^ (range 1.5–97). The baseline MRI characteristics of all 52 included patients are shown in Table 2. The changes in parameters between the baseline scan and the second scan in week 2 for the subgroup of 39 patients are given in Table 3. During the second week of CRT, all T2W-based tumor size parameters decreased significantly, while ADC mean significantly increased and ADC skewness decreased.

**Table 2:** Baseline MRI tumor characteristics for all included patients (*n* = 52) on baseline scan

	Baseline scan (*n* = 52)
–	*Median (range)*
Diameter (mm)	40.0 (14/140)
Volume (cm^3^)	14.5 (1.5/97.2)
EMTD (mm)	5.0 (0/28)
ADC mean	987 (797/1250)
ADC kurtosis	3.7 (2.4/9.3)
ADC skewness	0.6 (−0.7/1.9)
ADC SD	231.0 (121/372)
ADC entropy	4.2 (3.4/5.1)
–	*Total number*
EASI (number)	29/52

*ADC *apparent diffusion coefficient,* EMTD *extramural tumor depth, *EASI *external anal sphincter infiltration, *SD *standard deviation

**Table 3:** MRI tumor characteristics on baseline scan and second scan during week 2 of chemoradiotherapy in the patient subgroup (*n* = 39); Wilcoxon signed rank sum test

	Baseline scan (*n* = 39)	Second scan (*n* = 39)
–	*Median (range)*	
Diameter (mm)	40.0 (14/140)	30.0 (11/97)**
Volume (cm^3^)	14.9 (1.5/97.2)	6.7 (1.1/86)**
EMTD (mm)	5.0 (0/26)	3.0 (0/22)**
ADC mean	1010.0 (810/1250)	1210.0 (1000/1470)**
ADC kurtosis	3.7 (2.4/9.3)	3.6 (2.2/7.4)
ADC skewness	0.8 (0.7/1.9)	0.4 (−1.3/1.4)*
ADC SD	232.0 (121/372)	223.0 (105/387)
ADC entropy	4.2 (3.4/4.6)	4.2 (3.5/4.7)
–	*Total number*	
EASI (number)	24/39	19/39

*ADC *apparent diffusion coefficient,* EMTD *extramural tumor depth, *EASI *external anal sphincter infiltration, *SD *standard deviation

*significant (*p*  0.05), **highly significant (*p*  0.001)

**Fig. 2:**
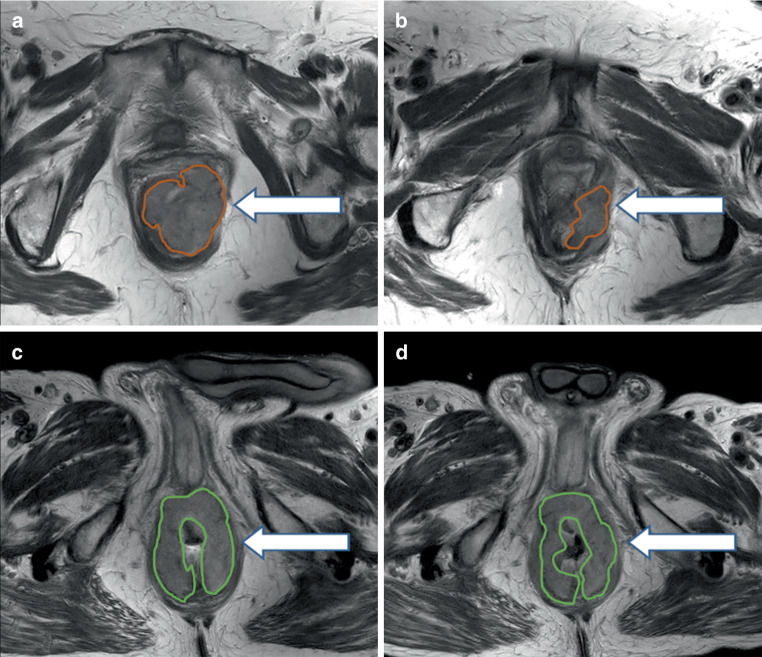
Transversal T2 image of a patient who had a 70% relative change in tumor volume and no recurrence: **a** Baseline scan, **b** second scan in week 2. Transverse T2 image of a patient who had a 7% relative change in tumor volume and recurrence: **c** baseline scan, **d** second scan in week 2. Tumor delineated with *coloured contours*. *White arrows* point to the delineated tumor

Patients in the subgroup (*n* = 39) who received two 3 T MRI scans (baseline scan and second scan) were divided into a without locoregional failure group (nonfailure; 32/39) and a locoregional failure group (failure; 7/32). There was no significant difference in the baseline characteristics and TNM staging between the groups. Differences in the relative change in the MRI characteristics from baseline to the second week scan between the groups are shown in Table 4. The decrease in tumor size was lower in the failure group, although the threshold for statistical significance was missed by a small margin. The results of logistic regression analysis to assess associations between MRI characteristics and their relative degree of change during CRT with locoregional failure are shown in Table 5. Small relative changes in the volume and diameter of the tumor between the scans were associated with treatment failure (Fig. 2), and this variable had the highest AUC (0.73 and 0.76, respectively). The optimal cutoff point for the relative change in tumor volume was −50%, and −12.5% for the relative change in tumor diameter (sensitivity 0.71, specificity 0.75).

**Table 4:** Relative changes in MRI tumor characteristics from baseline to the second week scan (*n* = 39) in the failure group versus the nonfailure group. Median differences and *p*-values were estimated by quantile regression

	Relative change		Median difference	*p*-value
	Failure group (*n* = 7)	Nonfailure group(*n* = 32)		
	Median, % (range)		% (CI)	
Diameter	−9.1 (−32.7/0.0)	−24.5 (−61.1/0.0)	16.6 (−0.9 to 34.0)	0.06269
Volume	−46.5 (−64.3/−7.3)	−62.5 (−90.4/−6.7)	15.4 (−9.7 to 40.4)	0.22212
EMTD	−41.7 (−100/0.0)	−25.0 (−100.0/200.0)	−16.7 (−78.3 to 45.0)	0.58726
ADC mean	20.0 (9.6/40.7)	18.7 (2.4/44.8)	1.3 (−12.3 to 15.0)	0.84704
ADC kurtosis	17.2 (−11.3/53.3)	1.7 (−63.5/69.1)	16.6 (−21 to 54.1)	0.37741
ADC skewness	5.4 (−124.3/450.0)	−34.4 (−201.4/1266.7)	33.9 (−83.1 to 151.0)	0.56061
ADC SD	−14.0 (−34/47.9)	−6.8 (−39.5/39.3)	−7.2 (−30.8 to 16.5)	0.54324
ADC entropy	−3.1 (−10.8/3.3)	3.0 (−10.5/22.0)	−7.0 (−17.6 to 3.6)	0.19022

*ADC *apparent diffusion coefficient,* EMTD *extramural tumor depth, *SD *standard deviation,* CI *external anal sphincter infiltration confidence interval

**Table 5:** Univariate regression models for the association of MRI characteristics before CRT (baseline scan), during week two of CRT (second scan) and their relative changes between the scans with locoregional failure

	Baseline (*n* = 52)	Week two (*n* = 39)	Relative change (*n* = 39)
*Volume*			
OR (95% CI)	1.01 (0.98 to 1.04)	1.03 (0.99 to 1.07)	1.04 (1.00 to 1.07)
AUC (95% CI)	0.651 (0.448 to 0.808)	0.701 (0.460 to 0.883)	0.732 (0.528 to 0.904)
*Diameter*			
OR (95% CI)	1.01 (0.98 to 1.05)	1.03 (0.98 to 1.08)	1.09 (1.0 to 1.18)
AUC (95% CI)	0.587 (0.276 to 0.844)	0.688 (0.414 to 0.882)	0.759 (0.518 to 0.924)
*EMTD*			
OR (95% CI)	1.02 (0.92 to 1.12)	1.00 (0.86 to 1.16)	0.99 (0.97 to 1.01)
AUC (95% CI)	0.568 (0.377 to 0.728)	0.464 (0.162 to 0.742)	0.326 (0.095 to 0.579)
*ADC mean*			
OR (95% CI)	1.00 (0.99 to 1.01)	1.00 (0.99 to 1.01)	1.01 (0.94 to 1.09)
AUC (95% CI)	0.527 (0.276 to 0.743)	0.482 (0.243 to 0.702)	0.549 (0.323 to 0.778)
*ADC skewness*			
OR (95% CI)	0.53 (0.14 to 1.97)	1.17 (0.30 to 4.53)	1.01 (0.99 to 1.00)
AUC (95% CI)	0.381 (0.203 to 0.616)	0.469 (0.227 to 0.742)	0.603 (0.278 to 0.848)
*ADC kurtosis*			
OR (95% CI)	0.49 (0.19 to 1.29)	0.96 (0.48 to 1.90)	1.02 (0.99 to 1.05)
AUC (95% CI)	0.330 (0.161 to 0.552)	0.536 (0.294 to 0.75)	0.688 (0.45 to 0.857)
*ADC SD*			
OR (95% CI)	1.01 (0.99 to 1.02)	1.00 (0.99 to 1.02)	1.00 (0.97 to 1.04)
AUC (95% CI)	0.594 (0.265 to 0.868)	0.5 (0.241 to 0.802)	0.455 (0.152 to 0.778)
*ADC entropy*			
OR (95% CI)	2.44 (0.23 to 26.33)	0.23 (0.01 to 3.85)	0.89 (0.78 to 1.01)
AUC (95% CI)	0.61 (0.354 to 0.833)	0.379 (0.181 to 0.594)	0.268 (0.114 to 0.447)

*CRT *chemoradiotherapy,* ADC *apparent diffusion coefficient,* OR *odds ratio, *AUC *area under the curve, *CI *confidence interval,* EMTD *extramural tumor depth, *SD *standard deviation

## Discussion

By conducting an additional early MRI examination during CRT, we have shown that T2W-based tumor size and the size-related characteristic EMTD decreased compared to the baseline scan. For ADC-based characteristics, skewness decreased, while the mean increased. Many of the baseline MRI characteristics were significantly correlated with each other, probably reflecting similar tumor features. None of the ADC-derived characteristics or EMTD and EASI were correlated with outcome, nor were any other MRI characteristics at baseline or during week 2 of CRT (second scan). The relative decrease in tumor size was lower in the treatment failure group, although the difference was not statistically significant and was probably related to the small number of patients.

For T staging in the UICC TNM classification, the greatest dimension of the tumor is used [30]. In our study, we additionally used T2W images to estimate tumor volume on a baseline MRI scan and found that the values were highly correlated with each other. In rectal cancer, EMTD is an important prognostic parameter [31]. We wanted to evaluate whether this metric could provide additional information on SCCA, being aware that rectal cancer and SCCA are two very different tumors despite of their anatomic neighborhood. Apart from its correlation with baseline tumor size, EMTD did not provide any additional information and was not correlated with treatment failure.

The majority of our study patients (39/52) underwent additional pelvic MRI during CRT. We chose week 2 as the timepoint for the early second scan according to previous studies on SCCA and squamous cell carcinoma of other sites [16, 19, 24, 25], enabling potential early changes in treatment plans during CRT.

Several MRI characteristics changed significantly between the two scans: tumor size and ADC skewness decreased while ADC mean increased. These changes probably reflect early tumor regression, most likely due to decreasing cellularity and heterogeneity, combined with increasing oedema. An increase in ADC mean and a decrease in tumor size during CRT are common findings for squamous cell carcinoma of other sites and SCCA [16, 17, 24, 25, 32]. Decreasing skewness in ADC histograms after CRT has also been reported in rectal cancer [33], metastatic ovarian cancer, and primary peritoneal cancer during chemotherapy [34].

The secondary aim of our study was to identify a response metric with the potential to predict outcome. Early identification of the subgroup of patients with treatment failure in the early phase during CRT would allow us to personalize treatment. Although there are no established alternative personalized and modified treatment concepts yet, one could consider, for instance, dose escalation, intensified treatment with chemotherapy, or immunotherapy for patients with a predicted risk for treatment failure.

None of the ADC-based histogram characteristics (mean, skewness, kurtosis, SD, entropy) or any of the baseline (*n* = 52) or week‑2 (*n* = 39) MRI characteristics correlated significantly with outcome in our study. Low relative changes in volume and diameter from the baseline scan to the second scan during CRT were associated with treatment failure, so these variables may represent easily assessable imaging-based biomarkers. This finding is in line with previous trials on SCC of other sites that reported that tumor size-based characteristics assessed on T2W MRI at different timepoints prior, during, or after CRT were correlated with outcome [18, 35], but such correlations have not yet been reported for SCCA.

Two previous studies on SCCA assessed the relative change in ADC mean between baseline scans and scans during CRT, but the results were different. Muirhead et al. [25] found that the median percentage change in ADC mean between baseline and week-2 scans was lower in the failure group, while Jones et al. [24] described no significant correlation of the relative change in ADC mean with local recurrence. In the latter study, several other features of the ADC histogram (baseline skewness and SD, week-2 skewness and SD, week-4 kurtosis and SD) were correlated with recurrence, in contrast to the results of our study. The results of the few existing studies for SCCA vary, and there is a need for further trials with more patients to assess ADC histogram-based characteristics. Future trials should also evaluate T2W images, as recent studies [22, 23, 36] found different first- or higher-order texture histogram characteristics on pretreatment T2W images to be correlated with outcome in SCCA.

One limitation of our study was the small sample size and the small number of locoregional treatment failures due to the rarity of SCCA. Future studies should focus on including larger numbers of patients in multicenter studies or meta-analyses. A feasible approach could be the use of distributed learning [37]. Another limitation is manual tumor delineation, which is a subjective process prone to intra- and interobserver variability. To improve the objectivity of tumor delineation, future studies should favor semiautomated methods (e.g., those using threshold ADC values) [38]. As DWI is especially prone to artifacts related to bowel gas and motion, future studies should therefore include both DWI and the more stable T2W-MRI. Finally, the choice of the timepoint for the second scan in the early phase (during week 2 of CRT) might not be ideal for SCCA, and future research should strive to identify the optimal timepoint for the second scan.

In conclusion, the relative change in tumor size between a baseline MRI scan prior to CRT and an early second scan during CRT might have potential as an easily assessable imaging-based biomarker for SCCA without the need to assess more complex MRI characteristics.
